# Supplementary material for: Integrated millimeter-wave cavity electro-optic transduction
Source: Nat Commun. 2026 Jan 6;17:1166. doi: 10.1038/s41467-025-67932-w (PMC12858801; doi:10.1038/s41467-025-67932-w)
Supplement: Supplementary file 1 — Supplementary Information [file 41467_2025_67932_MOESM1_ESM.pdf]

Supplementary Information: Integrated millimeter-wave cavity electro-optic transduction

Kevin K. S. Multani[1,2,3*†], Jason F. Herrmann[1,4*†], Emilio A. Nanni[3] and Amir H. Safavi-Naeini[1,4*]

[1]E.L. Ginzton Laboratory, Stanford University, Stanford, USA.
[2]Department of Physics, Stanford University, Stanford, USA.
[3]SLAC National Accelerator Laboratory, Stanford University, Menlo Park, USA.
[4]Department of Applied Physics, Stanford University, Stanford, USA.

*Corresponding author(s). E-mail(s): kmultani@stanford.edu; jfherrm@stanford.edu; safavi@stanford.edu;
[†]These authors contributed equally to this work.

Contents

S1 Device physics and theory **2**

S1.1 Nominal solutions of the equations of motion and practical considerations 2

S1.2 The electro-optic coupling rate and mm-wave electrode design considerations 5

S1.2.1 Deriving an expression for g_0 6

S1.2.2 Superconducting electrode design considerations for longitudinally varying fields . 6

S1.3 Input-output model of the mm-wave resonator in the presence of substrate modes 9

S1.4 Impact of substrate mode hybridization on the electro-optic conversion efficiency and sideband ratio 12

S2 Electro-optic coupling rate inference and related data analysis methodology **12**

S2.1 Optical and RF path calibrations 12

S2.2 Determining the intracavity pump photon number 13

S2.3 Determining the electro-optic conversion efficiency, η_{OE} 14

S2.4 The electro-optic coupling rate, g_0 as a function of optical power 15

S2.5 Determining the intracavity RF photon number 16

S2.6 The sideband ratio as a function of optical pump power 16

S3 Methodology for fitting the multiparameter substrate mode model **17**
S3.1 Temperature-dependent fitting procedure 18
S3.2 Optical-power-dependent fitting procedure 21

S4 Experimental Setup **22**
S4.1 Primary optical path and wavelength calibration 23
S4.2 Self-heterodyne measurement path 23
S4.2.1 Self-heterodyne measurement theory 25
S4.3 Primary RF path 27
S4.4 Optical-pump locking electronics path 29
S4.5 Images of packaging and cryostat 30

S5 Cryogenic photorefractive behavior and in-situ mitigation **31**

References **32**

S1 Device physics and theory

In this section, we describe the modeling of our device. We begin with the Hamiltonian first and solve the resulting equations of motion, derive an expression for the electro-optic interaction rate and discuss the electrode design, introduce a phenomenological model of the interaction between the substrate modes and the mm-wave cavity, and lastly describe the impact the substrate modes on the device's nominal performance.

S1.1 Nominal solutions of the equations of motion and practical considerations

We begin with the Hamiltonian of our cavity electro-optic system under the rotating wave approximation,

$$\hat{H}/\hbar = \hat{H}_0/\hbar + g_0\left(\hat{a}_+^\dagger \hat{a}_0 \hat{b} + \text{h.c.}\right) + g_0\left(\hat{a}_-^\dagger \hat{a}_0 \hat{b}^\dagger + \text{h.c.}\right), \tag{S1}$$

where,

$$\hat{H}_0/\hbar = \omega_{\text{RF}}\hat{b}^\dagger\hat{b} + \omega_-\hat{a}_-^\dagger\hat{a}_- + \omega_0\hat{a}_0^\dagger\hat{a}_0 + \omega_+\hat{a}_+^\dagger\hat{a}_+. \tag{S2}$$

Here $\hat{a}_+$, a_0, and $\hat{a}_-$ are the annihilation operators for the blue (ω_+), pump (ω_0), and red (ω_-) optical modes respectively; b is the annihilation operator for the mm-wave (ω_{RF}) mode; g_0 is the single-photon electro-optic coupling rate. In our experiment, all fields are coherent, so we displace each operator and neglect the fluctuations. Thus, we write the coupled mode equations of the classical amplitudes denoted by α's for the intracavity optical fields and β for the mm-wave field below. Note in our experiments, we

drive the mm-wave mode and the optical pump mode so we include input fields for the optical pump and the mm-wave modes, $|\alpha_{\text{in}}|^2 = P_0/(\hbar\omega_p)$, and $|\beta_{\text{in}}|^2 = P_{\text{RF}}/(\hbar\Omega)$,

$$\dot{\alpha}_0 = -(i\Delta_0 + \kappa_0/2)\alpha_0 - \sqrt{\kappa_{e,0}}\alpha_{\text{in}} \tag{S3}$$

$$\dot{\alpha}_+ = -(i\Delta_+ + \kappa_+/2)\alpha_+ - ig_0\alpha_0\beta \tag{S4}$$

$$\dot{\alpha}_- = -(i\Delta_- + \kappa_-/2)\alpha_- - ig_0\alpha_0\beta^* \tag{S5}$$

$$\dot{\beta} = -(i\Delta_{\text{RF}} + \kappa_{\text{RF}}/2)\beta - ig_0\alpha_+\alpha_0^* - ig_0\alpha_-^*\alpha_0 - \sqrt{\kappa_{e,\text{RF}}/2}\cdot\beta_{\text{in}}, \tag{S6}$$

where $\Delta_{\text{RF}} = \omega_{\text{RF}} - \Omega$, $\Delta_0 = \omega_0 - \omega_p$, $\Delta_+ = \omega_+ - (\omega_p + \Omega)$, and $\Delta_- = \omega_- - (\omega_p - \Omega)$; ω_p and Ω are the optical and mm-wave drive frequencies, respectively. Additionally, the mm-wave mode is modeled as being double-side coupled, which is why we have a factor of 1/2 for the input field coupling rate. Thus, the total linewidth is defined as $\kappa = \kappa_i + \kappa_e$ for all modes. In the steady-state, the intracavity field amplitudes are,

$$\alpha_0 = \frac{-\sqrt{\kappa_{e,0}}}{i\Delta_0 + \kappa_0/2}\cdot\alpha_{\text{in}} \tag{S7}$$

$$\beta = \frac{-\sqrt{\kappa_{e,\text{RF}}/2}}{i\Delta_{\text{RF}} + \kappa_{\text{RF}}/2 + \cancel{g_0^2|\alpha_0|^2\left(\frac{1}{i\Delta_+ + \kappa_+/2} + \frac{1}{i\Delta_- - \kappa_-/2}\right)}}\cdot\beta_{\text{in}} \tag{S8}$$

$$\alpha_+ = \frac{-ig_0}{i\Delta_+ + \kappa_+/2}\cdot\alpha_0\beta \tag{S9}$$

$$\alpha_- = \frac{-ig_0}{i\Delta_- + \kappa_-/2}\cdot\alpha_0\beta^*, \tag{S10}$$

where the diagonal strike-out denotes that we assume we are in the low-cooperativity limit. This is equivalent to assuming that there is no back-action on the optical modes from the dynamics of the mm-wave mode. Using the input-output boundary condition, $\alpha_k^{\text{out}} = \alpha_k^{\text{in}} + \sqrt{\kappa_{e,\text{k}}}\cdot\alpha_k$, and similarly $\beta^{\text{out}} = \beta^{\text{in}} + \sqrt{\kappa_{e,\text{RF}}/2}\cdot\beta$, we can compute the output fields,

$$\alpha_0^{\text{out}} = \left(1 - \frac{\kappa_{e,0}}{i\Delta_0 + \kappa_0/2}\right)\cdot\alpha_{\text{in}} \tag{S11}$$

$$\beta^{\text{out}} = \left(1 - \frac{\kappa_{e,\text{RF}}/2}{i\Delta_{\text{RF}} + \kappa_{\text{RF}}/2}\right)\cdot\beta_{\text{in}} \tag{S12}$$

$$\alpha_+^{\text{out}} = \frac{ig_0\sqrt{\kappa_{e,+}}}{i\Delta_+ + \kappa_+/2}\cdot\frac{\sqrt{\kappa_{e,0}}}{i\Delta_0 + \kappa_0/2}\cdot\alpha_{\text{in}}\cdot\beta \tag{S13}$$

$$\alpha_-^{\text{out}} = \frac{ig_0\sqrt{\kappa_{e,-}}}{i\Delta_- + \kappa_-/2}\cdot\frac{\sqrt{\kappa_{e,0}}}{i\Delta_0 + \kappa_0/2}\cdot\alpha_{\text{in}}\cdot\beta^*. \tag{S14}$$

With the solutions to the coupled mode equations, we compute two quantities that we use to estimate the g_0 of our device: the efficiency and the side-band ratio. We begin with the device efficiency in the low cooperativity limit,

$$\eta_{\mathrm{OE}} = \left| \frac{\alpha_+^{\mathrm{out}}}{\beta_{\mathrm{in}}} \right|^2 \tag{S15}$$

$$\approx g_0^2 \cdot \left(\frac{\kappa_{e,+}}{\Delta_+^2 + \kappa_+^2/4} \right) \left(\frac{\kappa_{e,\mathrm{RF}}/2}{\Delta_{\mathrm{RF}}^2 + \kappa_{\mathrm{RF}}^2/4} \right) \cdot n_{c,0} \tag{S16}$$

where $n_{c,0} = |\alpha_0|^2 = \kappa_{e,0}/(\Delta_0^2 + \kappa_0^2/4)|\alpha_{\mathrm{in}}|^2$ is the intracavity pump photon number. If we assume that $\omega_+ = \omega_0 + \omega_{\mathrm{RF}}$ and we drive on resonance $\omega_p = \omega_0$ with $\Omega = \omega_{\mathrm{RF}}$, then the expression simplifies to

$$\eta_{\mathrm{OE}} \approx 4C_0 \left(\frac{\kappa_{e,+}}{\kappa_+} \right) \left(\frac{\kappa_{e,\mathrm{RF}}}{2\kappa_{\mathrm{RF}}} \right) \cdot n_{c,0} \tag{S17}$$

Here $C_0 \equiv 4g_0^2/(\kappa_+ \kappa_{\mathrm{RF}})$ is the single-photon cooperativity.

The second quantity comes from the output sideband power ratio,

$$\Upsilon_{+,0} = \frac{P_+^{\mathrm{out}}}{P_0^{\mathrm{out}}} = \frac{\omega_p + \Omega}{\omega_p} \left| \frac{\alpha_+^{\mathrm{out}}}{\alpha_0^{\mathrm{out}}} \right|^2 \tag{S18}$$

$$\approx g_0^2 \cdot \left(\frac{\omega_p + \Omega}{\omega_p} \right) \cdot \frac{\left(\frac{\kappa_{e,+}}{\Delta_+^2 + \kappa_+^2/4} \right) \left(\frac{\kappa_{e,0}}{\Delta_0^2 + \kappa_0^2/4} \right)}{1 - 4\frac{\kappa_{e,0}(\kappa_0 - \kappa_{e,0})}{4\Delta_0^2 + \kappa_0^2}} \cdot n_{c,\mathrm{RF}}, \tag{S19}$$

where $n_{c,\mathrm{RF}} = |\beta|^2 = (\kappa_{e,\mathrm{RF}}/2)/(\Delta_{\mathrm{RF}}^2 + \kappa_{\mathrm{RF}}^2/4)|\beta_{\mathrm{in}}|^2$ is the intracavity mm-wave photon number. To simplify, we can assume the same as above (on-resonance), to arrive at the expression,

$$\Upsilon_{+,0} \approx 4C_0 \cdot \frac{\eta_{e,+}\eta_{e,0}}{(1 - 2\eta_{e,0})^2} \cdot \frac{\omega_+}{\omega_0} \cdot n_{c,\mathrm{RF}}, \tag{S20}$$

given that $\eta_e = \kappa_e/\kappa$ for each of the optical modes. Note, we can also compute the sideband ratio between the red and blue sidebands,

$$\Upsilon_{+,-} = \frac{\eta_{e,+}}{\eta_{e,-}} \cdot \frac{Q_+}{Q_-}. \tag{S21}$$

This equation predicts that if the red and blue modes have different quality factors that the observed peak power will be different. Indeed in Figure 3 of the main text, we observe the red and blue sidebands differ in the measured power, consistent with the above expression.

In our experiment, we also include the impacts of the substrate modes together with the above expressions of η_{OE} and $\Upsilon_{+,0}$ to compute an estimate of the electro-optic coupling rate g_0 (see Supplementary

Section S1.4). Calibration of optical and mm-wave path losses is important in computing both the on-chip efficiency $\eta_{\rm OE}$ and the sideband ratio $\Upsilon_{+,0}$. In the case of efficiency, we measure the output photon flux ($|\alpha_{+}^{\rm out}|^2$) via the optical spectrum analyzer and infer the incident mm-wave ($|\beta_{\rm in}|^2$) and intracavity optical photon number ($n_{c,0}$) through our calibrations. Therefore, the measured efficiency and on-chip efficiency are related by optical and mm-wave path losses,

$$\eta_{\rm OE}^{\rm meas} = \eta_{\rm output}^{\rm opt}\eta_{\rm input}^{\rm RF} \cdot \eta_{\rm OE}, \tag{S22}$$

where the output optical efficiency includes chip-fiber loss and path loss from the fiber inside of the cryostat to the optical spectrum analyzer, and the input mm-wave efficiency includes the respective path loss to the device. The mm-wave input efficiency includes the path loss of the mm-wave source from outside of the cryostat to right before the device and the loss reduction that comes from the increased conductivity of the waveguides inside the cryostat due to low-temperature operation. Importantly, $\eta_{\rm OE}$ also depends on the intracavity photon number in the optical pump mode, which is in part calculated from the input optical pump power and the input loss, $\eta_{\rm input}^{\rm opt}$. The optical input loss includes the path loss from the fiber directly outside of the cryostat to the fiber right before the fiber-chip interface and the fiber-chip loss. In the main text, we report $\eta_{\rm OE}$.

In the case of the sideband ratio, the optical input and output efficiencies do not enter the equation. This is because we directly measure the output power of the pump and the converted optical power on the optical spectrum analyzer; and both of these fields travel the same optical path, so path efficiencies are canceled in the ratio. Therefore, this is an optically self-calibrating measurement. Thus, the measured sideband ratio depends only on the mm-wave path loss,

$$\Upsilon_{+,0}^{\rm meas} = \eta_{\rm input}^{\rm RF} \cdot \Upsilon_{+,0}. \tag{S23}$$

Further details of our calculations and calibrations regarding device performance can be found in the Methods section of the main text, Supplementary Section S1.4, and Supplementary Section S2.

S1.2 The electro-optic coupling rate and mm-wave electrode design considerations

In the main text, we explain that the mm-wave superconducting resonator's electric field distribution must be unipolar across the TFLN interaction region. Here we present a brief discussion of the reason behind this, in addition to a derivation of the coupling rate.

S1.2.1 Deriving an expression for g_0

In systems similar to ours, the electro-optic coupling rate (g_0) can be derived from the nonlinear interaction energy arising from the $\chi^{(2)}$ nonlinearity,

$$\begin{aligned}\mathcal{H}_{\mathrm{NL}}^{(2)} \to \hat{\mathcal{H}}_{\mathrm{NL}}^{(2)} &= \frac{2\epsilon_0}{3}\hat{\mathbf{E}}\cdot\left(\chi^{(2)} : \hat{\mathbf{E}}\otimes\hat{\mathbf{E}}\right) \\ &= \frac{2}{3}\epsilon_0\sum_{k=1}^{3}\sum_{\ell=1}^{3}\sum_{m=1}^{3}\chi_{k\ell m}^{(2)}\hat{E}_k\hat{E}_\ell\hat{E}_m.\end{aligned} \tag{S24}$$

Expanding the electric field in terms of operators, $\hat{\mathbf{E}}(\mathbf{r},t) = \sum_{\alpha=1}^{4}\mathbf{E}^\alpha(\mathbf{r})\hat{a}_\alpha e^{-i(\omega_\alpha t - k_\alpha z)}$ + h.c. The indices represent the four modes of interest with bosonic annihilation operators, $\{\hat{a}_-, \hat{a}_0, \hat{a}_+, \hat{b}\} \equiv \{\hat{a}_1, \hat{a}_2, \hat{a}_3, \hat{a}_4\}$. Note that the component subscript i in, $E_i^\alpha(\mathbf{r})$, denotes the coordinate axes, $1 \mapsto x, 2 \mapsto y, 3 \mapsto z$. Writing out the tensor product and keeping only energy conserving terms (terms that obey the condition $|\omega_\pm - \omega_0| = \omega_{\mathrm{RF}}$, or, using the α-indices, $|\omega_{1,3} - \omega_2| = \omega_4$), we obtain the expression

$$\begin{aligned}\hat{E}_k\hat{E}_\ell\hat{E}_m = &\left[\hat{a}_+^\dagger\hat{a}_0\hat{b}\left(E_k^{+*}E_\ell^0E_m^{\mathrm{RF}} + E_\ell^{+*}E_m^0E_k^{\mathrm{RF}} + E_m^{+*}E_k^0E_\ell^{\mathrm{RF}}\right)e^{i(k_0+k_{\mathrm{RF}}-k_+)x} + \mathrm{h.c.}\right] \\ &+ \left[\hat{a}_-^\dagger\hat{a}_0\hat{b}^\dagger\left(E_k^{-*}E_\ell^0E_m^{\mathrm{RF}^*} + E_\ell^{-*}E_m^0E_k^{\mathrm{RF}^*} + E_m^{-*}E_k^0E_\ell^{\mathrm{RF}^*}\right)e^{i(k_0-k_{\mathrm{RF}}-k_-)x} + \mathrm{h.c.}\right].\end{aligned}$$

Comparing to Equation S1, and matching coefficients, we obtain an expression for the electro-optic coupling rate,

$$\hbar g_0 = \frac{2}{3}\epsilon_0 N_+ N_0 N_{\mathrm{RF}}\iiint_{\mathbb{R}^3}\boldsymbol{\psi}^{+*}\cdot\left(\chi^{(2)} : \boldsymbol{\psi}^0\otimes\boldsymbol{\psi}^{\mathrm{RF}}\right)e^{i(k_0+k_{\mathrm{RF}}-k_+)x}\mathrm{d}V. \tag{S25}$$

In the expression above, we have introduced unitless normalized spatial distributions, $\mathbf{E}^\alpha = N_\alpha\boldsymbol{\psi}^\alpha$. This normalization ensures if there is one photon in the mode, that the electric field energy is equal to $\hbar\omega_\alpha/2$. We can write the normalization constants in terms of the field mode volumes V_α as,

$$N_\alpha = \sqrt{\frac{\hbar\omega_\alpha}{2\epsilon_0 V_\alpha}} = \sqrt{\frac{\hbar\omega_\alpha}{2\epsilon_0}}\times\sqrt{\frac{1}{\iiint\boldsymbol{\psi}^{\alpha*}\cdot(\epsilon_r\boldsymbol{\psi}^\alpha)\mathrm{d}V}}. \tag{S26}$$

S1.2.2 Superconducting electrode design considerations for longitudinally varying fields

A large part of the engineering that goes into optimizing g_0 is the placement of the electrode with respect to the optical waveguides. In our device, the dominant nonlinearity is along the crystal axis ($\hat{\boldsymbol{z}}$-axis), corresponding to the nonlinearity $\chi_{333}^{(2)}$. We place electrodes (total length L_e) across one section of the optical racetrack resonator (total length L_o) to drive the electro-optic interaction. If we separate the RF field and the integral into longitudinal and transverse components, we can reveal the important design

considerations, i.e. $\psi_i^{\mathrm{RF}} = \psi_i^{t,\mathrm{RF}}(z,y)\psi_i^{\ell,\mathrm{RF}}(x)$ and $\iiint \mathrm{d}V = \iint \mathrm{d}A \int \mathrm{d}x$,

$$\hbar g_0 \approx 2\epsilon_0 N_+ N_0 N_{\mathrm{RF}} \times \Big(\iint_A \chi_{333}^{(2)} |\psi_3^0|^2 \psi_3^{t,\mathrm{RF}} \mathrm{d}A \Big) \times \Big(\int_0^{L_e} \psi_3^{\ell,\mathrm{RF}}(x) e^{i(k_0-k_+)x} \mathrm{d}x \Big). \tag{S27}$$

Equation S27 has three terms: (i) constants proportional to $1/\sqrt{V_\alpha}$ (V_α are the mode volumes of each field), (ii) an overlap integral of the transverse fields (we assume the two optical field distributions corresponding to the pump mode ω_0 and the sideband mode ω_+ are equivalent in our device) within the cross section of the TFLN, and (iii) an integration along the length of modulation, which includes a phase matching term. Because our mm-wave resonator is a standing wave resonator, we need to consider the longitundinally varying term carefully. In our case, the electrodes are much shorter than the optical racetrack, and we observe $(k_0 - k_+)x \approx 2\pi/L_o x \ll 1$ for $x \in [0, L_e]$. That is, the phase mismatch of neighboring optical modes in a racetrack resonator is minimal compared to the phase mismatch that arises from the longitudinally varying RF field.

Now, consider the simplest electrode design: two metal slabs, one placed above and one placed below the TFLN waveguide, in a coplanar stripline geometry. The amplitude of the RF field between these electrodes is $\psi_3^{\ell,\mathrm{RF}} = \Psi$. In this scenario, the electrodes can be open-ended or close-ended on one side i.e. a half-wave or quarter-wave coplanar stripline resonator. In the case of the half-wave resonator the wavenumber for the fundamental mode is $k_{\mathrm{RF}} = \pi/L_e$, therefore phase matching integral can be written as,

$$I = \int_0^{L_e/2} \Psi e^{i\pi x/L_e} \mathrm{d}x - \int_{L_e/2}^{L_e} \Psi e^{i\pi(x-L_e/2)/L_e} \mathrm{d}x = 0. \tag{S28}$$

The phase term is split into two regions because at $x = L_e/2$ the polarity of the electric field goes from parallel to antiparallel with respect to the crystal axis. So, in the case of a simple half-wave resonator, the electro-optic coupling rate vanishes.

For the quarter-wave resonator, the rate does not vanish because the field does not change polarity. However, the electrode would need to cross-over the TFLN waveguide, which introduces loss for the optical modes (due to proximity to the metal) and loss for the RF mode (due to proximity to the oxide cladding atop the optics). Therefore, for the device presented in the main text, we start with a half-wave resonator and modify the electrodes so that the RF field distribution across the TFLN is more approximate to a quarter-wave resonator.

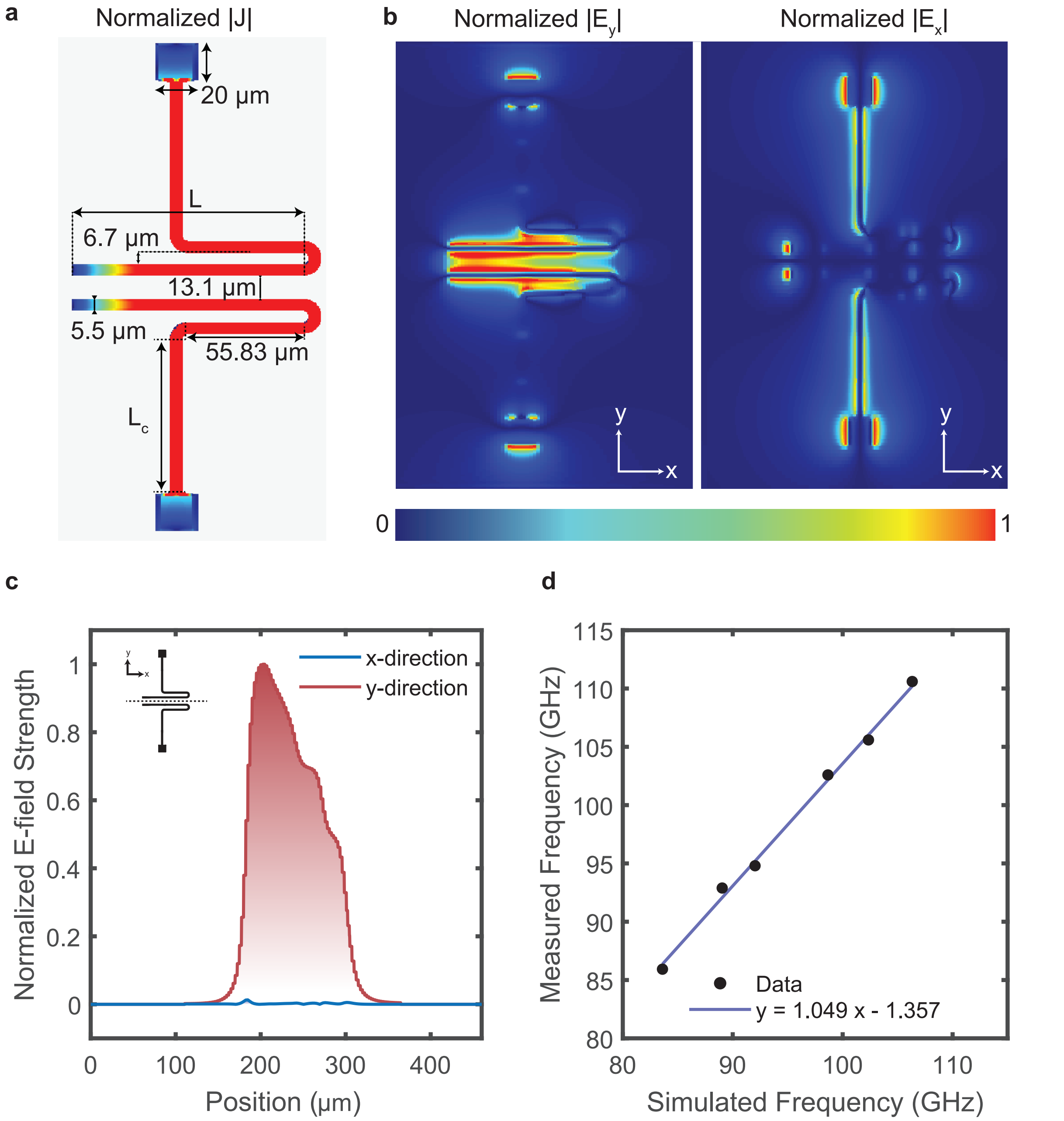

Supplementary Fig. 1 Millimeter-wave cavity design. **a** SONNET-simulated [7] normalized current density and geometry of the mm-wave electrodes of the device shown in the main text (on resonance). The device in the main text has $L = 111.65\,\mu\text{m}$ and $L_c = 82\,\mu\text{m}$. **b** SONNET simulation of the normalized electric field magnitudes in the x and y direction of the mm-wave electrodes (on resonance). **c** A line-cut of the data in sub-figure (b), showing the electric field distribution along the line-cut. The location of the line-cut is shown in the inset on the top-left. **d** Data of simulations and measurements of the NbTiN-witness resonators. We used this data to design the NbTiN electrode geometry of the device in the main text. To vary the frequency, we only changed the length of the straight section L.

S1.3 Input-output model of the mm-wave resonator in the presence of substrate modes

As described in the main text, the mm-wave spectrum contains a cacophony of substrate-supported modes that couple to the mm-wave superconducting resonator. We are able to probe the mm-wave mode via a dipole interaction, just like an antenna. However, we posit that the electric field supported by the resonator can also couple to otherwise inaccessible (dark) modes within the Sapphire substrate. In this section, we derive a model to describe this situation.

Suppose there are N total substrate modes, each with a coupling rate J_n to the mm-wave mode, total linewidth γ_n, and resonant frequency ω_n. The coupled mode equations of the classical amplitudes in a frame rotating with the mm-wave drive frequency Ω, can be written as,

$$\dot{\beta} = -\left(i\Delta_{\text{RF}} + \frac{\kappa_{\text{RF}}}{2}\right)\beta - i\sum_{n=1}^{N} J_n^* s_n - \sqrt{\frac{\kappa_{e,\text{RF}}}{2}}\beta_{\text{in}} \tag{S29}$$

$$\dot{s}_n = -\left(i\Delta_n + \frac{\gamma_n}{2}\right)s_n - iJ_n\beta, \tag{S30}$$

where, s_n is the nth substrate mode intracavity amplitude, $\Delta_{\text{RF}} = \omega_{\text{RF}} - \Omega$, and $\Delta_n = \omega_n - \Omega$. Note the mm-wave resonator is double-side coupled, necessitating the factor of two in the drive term, just as in the above section. In steady-state, the mm-wave intracavity amplitude is,

$$\beta = \frac{-\sqrt{\kappa_{e,\text{RF}}/2}}{i(\Delta_{\text{RF}} + \tilde{\delta}) + (\kappa_{\text{RF}} + \tilde{\gamma})/2} \cdot \beta_{\text{in}}. \tag{S31}$$

where,

$$\tilde{\delta} = -\sum_{n=1}^{N} \frac{|J_n|^2\Delta_n}{\Delta_n^2 + \gamma_n^2/4} \tag{S32}$$

$$\tilde{\gamma}/2 = \sum_{n=1}^{N} \frac{|J_n|^2\gamma_n/2}{\Delta_n^2 + \gamma_n^2/4}. \tag{S33}$$

Using the input-output boundary condition $\beta_{\text{out}} = \beta_{\text{in}} + \sqrt{\kappa_{e,\text{RF}}/2} \cdot \beta$, we can write the mm-wave output field amplitude as,

$$\beta_{\text{out}} = \underbrace{\left(1 - \frac{\kappa_{e,\text{RF}}/2}{i(\Delta_{\text{RF}} + \tilde{\delta}) + (\kappa_{\text{RF}} + \tilde{\gamma})/2}\right)}_{\text{transmission coefficient, } S_{21}} \cdot \beta_{\text{in}}. \tag{S34}$$

Here we see that coupling to the substrate modes is indistinguishable from a shift in detuning and an increased linewidth of the superconducting mm-wave mode. As a result, the intracavity population

of the mm-wave mode can be affected, which impacts the device performance (see next section). Thus, carefully fitting and understanding the substrate mode parameters is needed. We provide details of our fitting methodology in Supplementary Section S3.

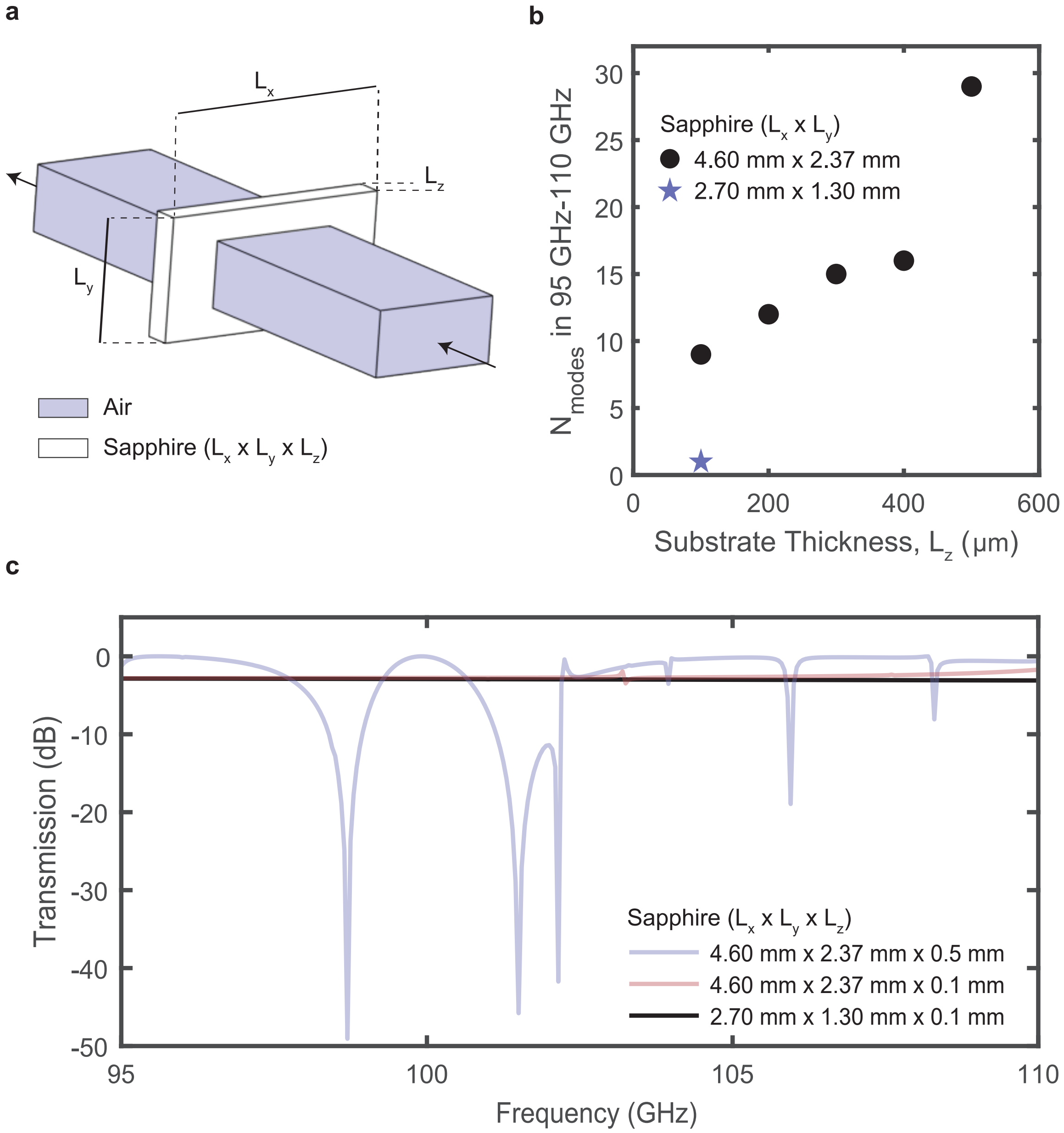

Supplementary Fig. 2 Simulations of substrate mode behavior as a function of chip geometry. **a** Geometry of the COMSOL [6] simulation, with labels. The input and output waveguide ports are labeled by arrows, and all boundaries are set to be perfect electrical conductors. **b** Number of modes supported by the substrate in the geometry defined in subfigure (a) within the frequency range of interest. The number of modes was given by COMSOL via eigenfrequency simulations. Black dots indicate a substrate with the same dimensions as our sample (top-right-most datapoint), and the reduction in modes as the substrate thickness is reduced. The blue star indicates a dimensionality that should support minimal substrate modes in our target frequency range. **c** COMSOL simulations of the transmission spectrum through the structure in subfigure (a), revealing resonances supported by the Sa substrate. Reducing the dimensionality drastically suppresses the number of supported substrate modes in our target frequency range in this coupling geometry.

S1.4 Impact of substrate mode hybridization on the electro-optic conversion efficiency and sideband ratio

In this section, we discuss the implications of the substrate modes on the nominal device performance as discussed in Supplementary Section S1.1. As shown in the previous section, coupling to substrate modes impacts the mm-wave intracavity population by adding an additional shift to the mm-wave resonance detuning and total linewidth. Thus, the on-chip efficiency of the transduction process and the sideband ratio are modified according to,

$$\eta_{\mathrm{OE}} \approx g_0^2 \cdot \left(\frac{\kappa_{e,+}}{\Delta_+^2 + \kappa_+^2/4} \right) \cdot \left(\frac{\kappa_{e,\mathrm{RF}}/2}{(\Delta_{\mathrm{RF}} + \tilde{\delta})^2 + (\kappa_{\mathrm{RF}} + \tilde{\gamma})^2/4} \right) \cdot n_{c,0}, \tag{S35}$$

$$\Upsilon_{+,0} \approx g_0^2 \cdot \left(\frac{\omega_p + \Omega}{\omega_p} \right) \cdot \left(\frac{\kappa_{e,+}}{\Delta_+^2 + \kappa_+^2/4} \right) \cdot \left(\frac{\frac{\kappa_{e,0}}{\Delta_0^2 + \kappa_0^2/4}}{1 - 4\frac{\kappa_{e,0}(\kappa_0 - \kappa_{e,0})}{4\Delta_0^2 + \kappa_0^2}} \right) \cdot n_{c,\mathrm{RF}}, \tag{S36}$$

where,

$$n_{c,\mathrm{RF}} = |\beta|^2 \tag{S37}$$

$$= \frac{\kappa_{e,\mathrm{RF}}/2}{(\Delta_{\mathrm{RF}} + \tilde{\delta})^2 + (\kappa_{\mathrm{RF}} + \tilde{\gamma})^2/4} \cdot \frac{P_{\mathrm{RF}}}{\hbar\Omega}. \tag{S38}$$

We use these equations for our estimation of g_0 in the main text, with further details on the fitting procedure in Supplementary Section S2.

S2 Electro-optic coupling rate inference and related data analysis methodology

In this section, we provide details on the procedures used to estimate the electro-optic coupling rate g_0. As discussed in the main text and previous supplementary sections, we infer g_0 using two different quantities: the on-chip transduction efficiency (Eq. S35); and the ratio of the converted sideband power and the pump power (Eq. S36).

S2.1 Optical and RF path calibrations

We begin by describing how we calibrate path losses. As mentioned in Supplementary Section S1.1, the measured efficiency includes both optical and RF path losses. For the RF path, we calculate the power incident to the chip,

$$|\beta_{\mathrm{in}}|^2 = \eta_{\mathrm{input}}^{\mathrm{RF}} |\beta_{\mathrm{source}}^{\mathrm{RF}}|^2, \tag{S39}$$

where $|\beta_{\mathrm{source}}^{\mathrm{RF}}|^2 = P_{\mathrm{source}}^{\mathrm{RF}}/(\hbar\Omega)$. By using a diode-based power detector (Pacific Millimeter-wave Products), we measure the power of the RF extender to be $P_{\mathrm{source}}^{\mathrm{RF}} = 1.87\,\mathrm{mW}$ at $\Omega = 2\pi \cdot 105.285\,\mathrm{GHz}$. This frequency is associated with the peak transducer response. The same detector is used to measure the insertion loss from the source to the device at room temperature. We measure this path loss to be -21.2 dB. Notably, the portion of the RF path that is thermalized to 4 K exhibits reduced loss at cryogenic temperatures, due to increased conductivity of the WR10 waveguides. We measure this cryogenic "gain" to be 3.56 dB. Therefore, the total RF path loss up to the device at cryogenic temperatures is -17.6 dB or $\eta_{\mathrm{input}}^{\mathrm{RF}} = 1.72\%$. More details on the cryogenic calibration procedure can be found in [1].

For the optical path, we consider transmission through three distinct regions: the input path just before the cryostat; the path through the cryostat; and the output path from the cryostat to the OSA. We directly measure the power incident to the cryostat via a calibrated 99:1 beam splitter. To measure the insertion loss through the cryostat, we optimize the optical polarization in the vicinity of the three modes we care about. However, we cannot disambiguate the insertion loss of the input grating coupler and the output grating coupler; so the best we can do is assume the loss is equal. For the measurements in this work, we find the single-sided grating coupler efficiency to be $\eta_{\mathrm{input}}^{\mathrm{opt}} = 8.84\%$. Lastly, the optical path loss between the cryostat output and the OSA is 82.3%. This gives a total output efficiency of $\eta_{\mathrm{output}}^{\mathrm{opt}} = 7.28\%$.

S2.2 Determining the intracavity pump photon number

We plot the intracavity pump photon number as a function of optical power (in the waveguide) in Supplementary Figure 3a. The "y-axis" of this plot depends on the precise detuning of our pump tone from the pump mode resonance frequency, ω_0. We determine this detuning for each input pump power by matching our measured transmission through the cavity with the theoretical transmission. As described in the main text methods section, the following relation holds for the transmission through the optical resonator,

$$T = \frac{P_{\mathrm{OSA}}}{P_{\mathrm{PM}}} \cdot \eta_0 \tag{S40}$$

$$= \left|1 - \frac{\kappa_{e,0}}{i\Delta_0 + \kappa_0/2}\right|^2. \tag{S41}$$

P_{OSA} and P_{PM} are the optical pump power measured on the OSA (at the end of the measurement chain) and on a power meter (before sending the laser tone into the cryostat), respectively, and η_0 encapsulates optical path losses and calibrations on both the input and output paths. We measure every value in the second equality through our self-heterodyne characterization (see main text), except for the pump detuning, $\Delta_0 = \omega_0 - \omega_p$. By equating these two expressions, we can solve for the detuning Δ_0. We

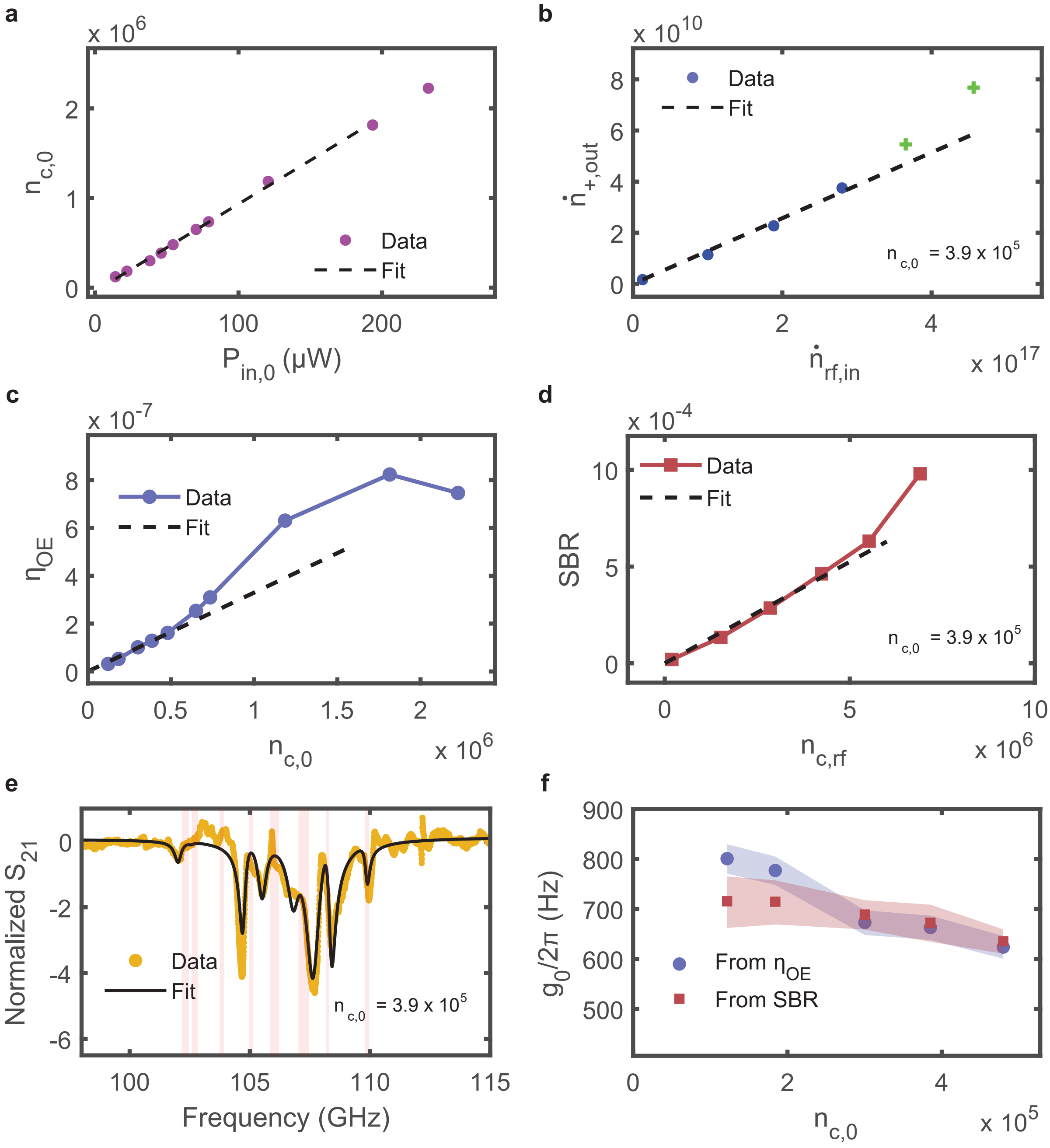

Supplementary Fig. 3 Step-by-step data analysis required to extract the electro-optic coupling. **a** A calibrated plot of the intracavity pump photon number versus on-chip optical pump power. The data is nearly linear, with variation coming from differences in the lock-point detuning of the pump tone from resonance. **b** A representative example of sideband photon flux plotted against RF photon flux. At low RF powers, this ratio is linear and can be fit to determine $\eta_{\rm OE}$. **c** Reproduction of the main text Fig. 3b. Each datapoint is determined by fitting $\eta_{\rm OE}$ as in (b). **d** Reproduction of main text Fig. 3c. **e** Representative example of RF spectrum and fit at a particular optical pump power. The red vertical bars are centered on substrate mode frequencies with widths matching the 3-dB linewidths of the modes. **f** Reproduction of main text Fig. 3e.

then compute the intracavity pump photon number, $n_{c,0} = \kappa_{e,0}/(\Delta_0^2 + \kappa_0^2/4) \cdot \frac{P_0}{\hbar\omega_p}$. We repeat this process for every datapoint in Supplementary Fig. 3a, thereby calibrating the "x-axis" of main text Fig. 3b.

S2.3 Determining the electro-optic conversion efficiency, $\eta_{\rm OE}$

In this section, we provide our methodology to determine the electrical-to-optical transduction efficiency. For each applied pump power (each datapoint in Supplementary Fig. 3a), we vary the applied RF

modulation power. For each applied RF power, we vary the RF modulation frequency. At each frequency, we measure an OSA trace, such as that given in main text Fig. 3a. Then, we determine the OSA trace that yields the greatest optical sideband power, which we assume to be the experimental condition that satisfies the frequency matching between the RF modulation and the optical pump-to-sideband-mode frequency spacing. From these data we estimate the on-chip sideband photon flux, given our calibrations (discussed above). We also compute incident RF photon flux given the applied RF power, thereby obtaining a list of $\dot{n}_{+}^{\text{out}}$ v.s. $\dot{n}_{\text{RF}}^{\text{in}}$. An example of these data is plotted in Supplementary Fig. 3b. By fitting a line to the lower-power datapoints in Supplementary Fig. 3b, we determine the electrical-to-optical conversion efficiency. Measuring in this way allows us to average over fluctuations or errors in the value, which might enter the measurement at individual datapoints. We repeat this process for every optical pump power we apply to the device. For each optical power, we then plot the efficiency, thereby obtaining main text Fig. 3b (re-plotted as Supplementary Fig. 3c).

The pseudo-code/algorithm to reproduce Supplementary Fig. 3c is as follows (indents denote for-loop level, as in Python):

1. For each optical pump power from P_1^{opt} to P_n^{opt}:
   (a) For each RF modulation power from P_1^{RF} to P_m^{RF}:
      (i) For each RF modulation frequency from Ω_1 to Ω_l:
         (A) Measure the optical sideband spectrum on the OSA (see Fig. 3a, in the main text).
      (ii) Identify the spectrum with the greatest optical sideband power and record both the pump and sideband powers.
      (iii) Infer the on-chip optical photon flux and RF photon flux using the path loss calibrations given in Section S2.1.
   (b) Plot and linearly fit $\dot{n}_{+}^{\text{out}}$ v.s. $\dot{n}_{\text{RF}}^{\text{in}}$. The slope of this line is η_{OE}.

S2.4 The electro-optic coupling rate, g_0 as a function of optical power

Here we explain how we obtain an estimate for the electro-optic coupling rate as a function of optical power. In principle, these two quantities are independent of one another. However, since the mm-wave cavity parameters (i.e., frequency and linewidth) depend on optical power, we can get multiple estimates of g_0 (one for each applied optical power). In Fig. 3b of the main text, reproduced as Supplementary Fig. 3c, we see that the first five datapoints in this plot grow linearly with intracavity photon number, as expected from theory. We obtain the slope of this linear region and, by incorporating the optical power

dependence of our mm-wave cavity mode (Equation S35), we infer five separate values of g_0 (see Supplementary Fig. 3f). More details on the fitting procedure to obtain the mm-wave cavity parameters is found in Section S3.

S2.5 Determining the intracavity RF photon number

The RF photon cavity occupation is a function of temperature, optical power, and its coupling to the surrounding substrate modes. This quantity is directly used in inferring the g_0 from the sideband ratio, $\Upsilon_{+,0}$ (see Eq. S36 and section below). Experimentally, we measure the RF spectrum for each optical pump power and RF power. We fit the low-RF-power trace at each optical power, taking into account the various substrate modes. The linewidths and frequencies of these substrate modes are previously determined from fitting the RF spectrum at various cryostat temperatures (as explained in the main text and in Supplementary Section S3). Therefore, with the substrate mode parameters fixed, each fit determines only the superconducting mm-wave resonance frequency and linewidth at that particular applied optical power. In Supplementary Fig. 3e we depict one such trace and fit; the center frequency and width of the red vertical bars denote the substrate mode frequencies and linewidths, respectively. The mode parameters from the fit are then used to determine the intracavity RF population using Eq. S31.

S2.6 The sideband ratio as a function of optical pump power

As with the electro-optic coupling rate, the sideband ratio (SBR) is, in principle, uncorrelated with the optical pump power. In practice, however, the SBR depends on optical pump power due to the optical power dependence of the mm-wave cavity parameters. The sideband ratio (SBR) is defined as the ratio between the transmitted power in the blue sideband frequency versus the transmitted power at the optical pump frequency. For a given optical pump power, we compute the SBR for each of the applied RF modulation powers. A reproduction of this plot from main text Fig. 3c is given in Supplementary Fig. 3d. We fit a line to the linear regime of this plot (corresponding to low RF modulation powers). This line has a slope proportional to g_0^2 (see Eq. S36). Similar to η_{OE} (see above), we solve for g_0 from this slope, accounting for the shifted superconducting RF mode and substrate modes separately for each datapoint. A reproduction of main text Fig. 3e is given in Supplementary Fig. 3f. This figure presents the values of g_0 determined from both the SBR and η_{OE}-fitting methods.

S3 Methodology for fitting the multiparameter substrate mode model

In our experiment, we observe dips in the RF spectrum that we associate with substrate modes. These are high-frequency electromagnetic modes that are resonant in the approximately 500 µm-thick Sa substrate of our device. The presence of these modes is evidenced by simulations (see Methods), and we derive a model describing their interaction with the superconducting (SC) mm-wave mode in Supplementary Section S1.3. Importantly, if there is overlap between the electromagnetic field of the mode in the SC resonator and the field of the substrate mode, the two modes will couple, leading to an effective detuning imparted on the SC resonator mode frequency, and a broadening of its linewidth (inducing an effective loss of mm-wave photons from the SC resonator). Our model (see Eqs. S29-S34) and our fitting of this model to our data, adheres to the following assumptions:

1. The substrate modes are *only* excited through hybridization with SC resonator mode; they cannot be driven directly by the impinging RF field inside the WR10 waveguide.
2. Both the total loss rate (κ_{RF}) and the coupling loss rate ($\kappa_{e,\mathrm{RF}}$) of the SC mode can only increase as temperature increases.
3. The SC resonator frequency can only decrease ("red-shift") with increasing temperature.

Note that the temperature can be increased by both changing the temperature inside the cryostat thermally, or by pumping enough light into the optical resonator to locally heat the SC resonator. In order to determine the temperature dependence and parameters of the SC mm-wave mode and the substrate modes, we independently sweep the cryostat temperature (with optical power off) and record the RF spectra, and sweep the incident optical power to the chip (with the cryostat at base temperature), and record the RF spectra. We then fit the recorded spectra as described in the following sections. We also tested the results of fitting with a modified assumption "2" restricting the coupling loss rate, $\kappa_{e,\mathrm{RF}}$ to *only decrease* (while the total loss rate is still restricted to only increase). In this case, we found our predictions for η_{OE} and g_0 do not vary much from the reported values and are within the reported confidence intervals. Therefore, we report the results in this manuscript using the original assumption "2" given above.

S3.1 Temperature-dependent fitting procedure

Using particle swarm optimization (PSO), we iteratively fit the temperature-swept RF data to infer the parameters of each substrate mode. We first identify eight substrate modes that couple to the SC resonance as it thermally tunes. Our model for RF transmission, S_{21}^{RF} is then given by equation S34. We also include a linear background that is allowed to vary for each spectrum, yielding an expression comprising 29 free parameters: the SC mode parameters κ_{RF}, $\kappa_{e,\mathrm{RF}}$, Δ_{RF}; the substrate mode parameters γ_n, ω_n, J_n for $n \in \{1, 2, ..., 8\}$; and the linear background slope m and intercept b. The full expression is then:

$$S_{21}^{\mathrm{RF}} = \left(1 - \frac{\kappa_{e,\mathrm{RF}}/2}{i(\Delta_{\mathrm{RF}} + \tilde{\delta}) + (\kappa_{\mathrm{RF}} + \tilde{\gamma})/2}\right) + m\Omega + b \tag{S42}$$

where $\tilde{\delta}$ and $\tilde{\gamma}$ are given by equations S32 and S33 respectively, with $\Delta_n = \omega_n - \Omega$.

We repeatedly fit the temperature-dependent spectra and average over the results to fix additional parameters of these modes, thereby reducing the number of parameters that subsequent iterations of the PSO must optimize. Our detailed procedure follows:

1. Fit four RF spectra at the temperatures 4.89 K, 6.00 K, 6.70 K, and 7.01 K, normalized to a high-temperature background spectrum (at 13.01 K). Identify eight common substrate modes across these spectra.
2. Iteratively fit $|S_{21}^{\mathrm{RF}}|^2$ over 22 temperatures ranging from 4.89 K to 6.90 K, allowing all parameters to vary. After fitting all 22 spectra, we average over the fit results for the frequencies of the substrate modes, ω_n. We only include fit results in the averaging for which the fit of that particular substrate mode looks reasonable. An example comparing such fits is given in Supplementary Fig. 4.
3. Fix some of the frequencies and repeat step (2) until all ω_n are fixed.
4. Repeat the procedure in steps (2)-(3), but now with fixed frequencies, ω_n and allowing only the coupling rates J_n and loss rates γ_n to vary. As before, average over all reasonable fits to fix the γ_n of each mode.
5. Lastly, fix both ω_n and γ_n of all substrate modes. Fit all 22 temperature spectra, allowing only J_n, the linear background, and the SC mode parameters to vary, thereby obtaining how ω_{RF} and κ_{RF} tune with temperature.

Examples of fits from this process are given in Supplementary Fig. 4. These examples demonstrate which types of fits would be included in the average of the frequency (or comparably the loss rate) for a given substrate mode.

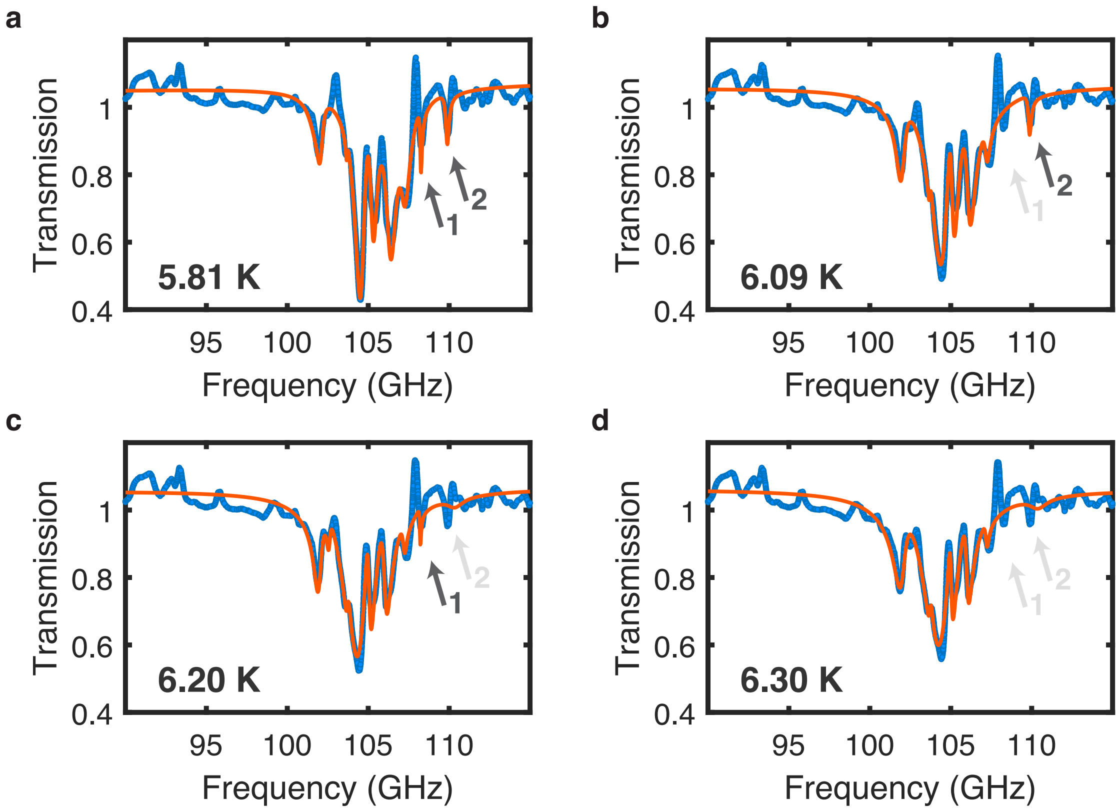

Supplementary Fig. 4 RF spectrum fits at four different temperatures. Transmission is normalized to a high-temperature (non-superconducting) background spectrum. Blue is data, and red is the resulting fit from PSO optimization. Dark grey arrows indicate two example substrate modes “1” and “2.” **a** RF spectrum taken at platform temperature $\sim$ 5.81 K. The dark grey arrows indicate that the fit results from this spectrum would be included in the average to determine the frequencies of both modes “1” and “2.” **b** RF spectrum taken at platform temperature $\sim$ 6.09 K. The dip corresponding to substrate mode “1” is missing from the fit, so this result would not be included in the average to determine the frequency of mode “1.” However, the dip corresponding to mode “2” is still reasonable, so we would include this result when averaging for the frequency of mode “2.” **c** RF spectrum taken at platform temperature $\sim$ 6.20 K. This is the opposite situation to (b). The fit for mode “1” is reasonable, while the fit for mode “2” is poor. We would only include this result in the average to determine the frequency of mode “1,” but not for the frequency of “2.” **d** RF spectrum taken at platform temperature $\sim$ 6.30 K. In this case neither dip is present, so we would not include the results of this fit in the averaging to determine either mode “1” or “2.”

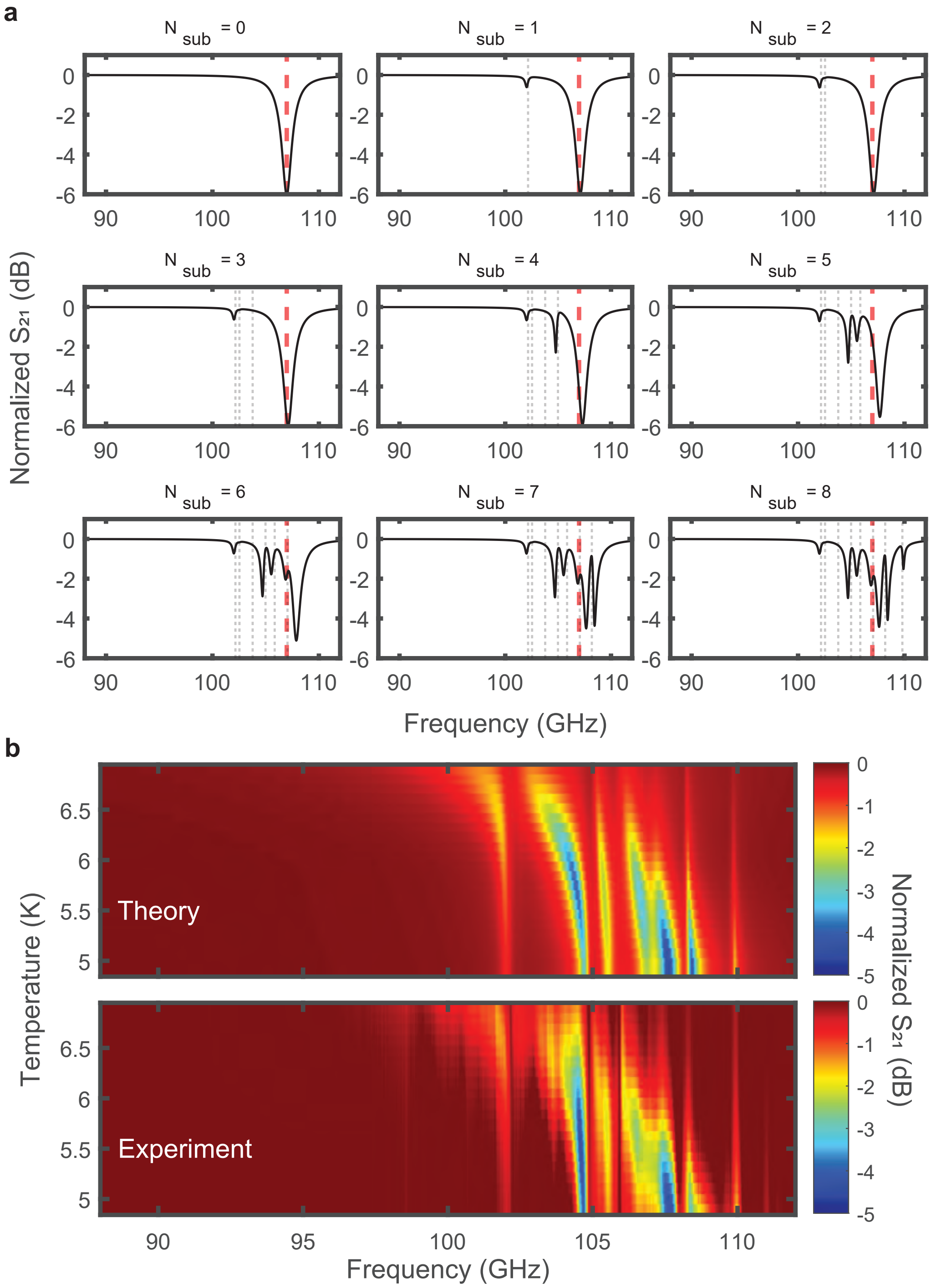

Supplementary Fig. 5 Impact of substrate modes on the millimeter-wave spectrum as a function of number of substrate modes and temperature. **a** The modeled millimeter-wave transmission spectrum (see Eq. 6 in the main text) at 4.9 K, as we incrementally add substrate modes into the model. The grey-dashed lines indicate the bare substrate mode frequencies and the red dashed lines show the bare millimeter-wave mode frequency (see Table 1 in the main text). **b** The theoretical (top) and experimental (bottom) temperature dependence of the millimeter-wave transmission spectrum. In the theory plot, we use our fitted values of the superconducting millimeter-wave cavity as a function of temperature and plot Eq. 6 in the main text, assuming the substrate mode parameters and couplings are constant.

S3.2 Optical-power-dependent fitting procedure

After identifying ω_n and γ_n for all substrate modes, we repeat step (5) of the above procedure, but over optical-power-dependent spectra at a fixed RF power. Using only low-RF-power datasets, we allow the SC resonance parameters to vary, along with the coupling rate, J_n ($n \in [1, \ldots, 8]$) of the eight substrate modes, and the linear background of each spectrum. From these fits, we identify how the SC mode frequency and loss rate tune with increasing optical power.

S4 Experimental Setup

In this section we go into detail for each part of the experimental setup. The complete setup is shown in Supplementary Figure 6.

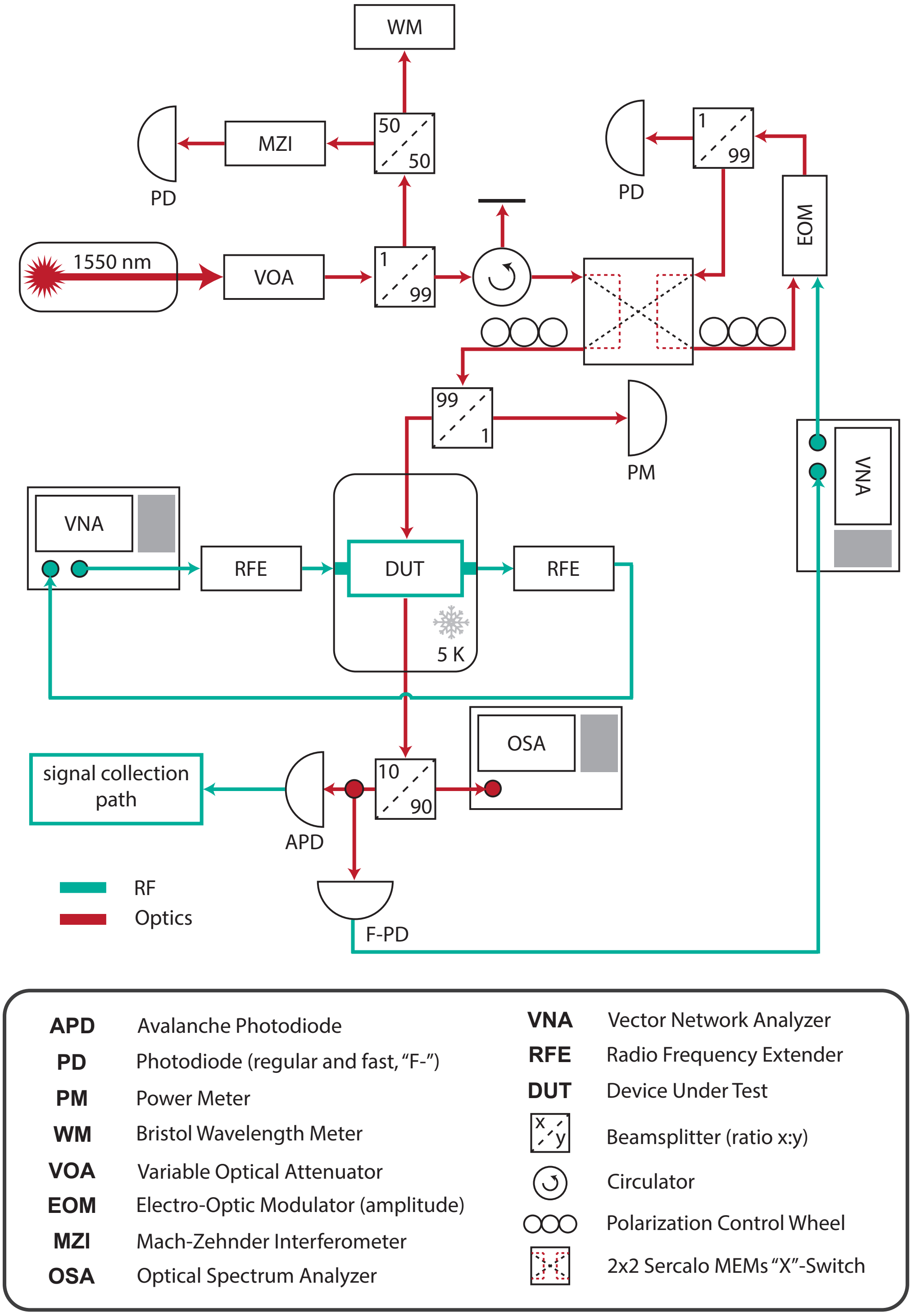

Supplementary Fig. 6 Complete measurement schematic depicting all paths and equipment used for the experiment. Red arrows denote optical paths, while aqua arrows denote RF paths. The device under test (DUT) is held within a cryostat at $T \approx 4.9\,\mathrm{K}$.

S4.1 Primary optical path and wavelength calibration

The optical path used for characterizing the on-chip mm-wave modulation and optical sideband generation is shown in Supplementary Figure 7. Light from a telecom laser (Santec TSL-710) is first attenuated via a MEMs variable optical attenuator (VOA). It then passes through a beam splitter, with most of the light continuing to the cryostat (Montana Instruments S200 Cryostation). A fraction of the light is passed into a wavelength calibration sub-path.

In the calibration sub-path, the light is split with a $50:50$ beamsplitter, with half passed to a wavemeter (WM, Bristol 621 Wavelength Meter) for wavelength measurement, and the other passed through a Mach-Zehnder interferometer (MZI), with a measured FSR of ~ 325 MHz). While collecting optical spectra of our device, we record the initial wavelength of the laser on the WM and then sweep the laser, simultaneously recording transmission through the device (DUT) and the fringing from the MZI. By combining the WM reading with the measured MZI spectrum, we can calibrate the wavelength at each point in the recorded device spectrum. While there is some error in the exact wavelength reported by the WM, the calibrated wavelength spacing between recorded data points is much more accurate, ensuring confident measurements of wavelength *ranges*, such as the optical mode linewidths and the free spectral range (FSR) of the racetrack resonator.

In the primary path, light passes through a circulator (to suppress reflections), followed by a 2x2 “x-switch” (Sercalo, SL2x2). With the switch in a bar configuration, the optical signal bypasses the EOM self-heterodyne sub-path (see Section S4.2), continues through another beamsplitter (used for optical power calibration), and is incident to the DUT inside the cryostat. The cryostat is maintained at nearly 5 K for the duration of measurements in this paper. The output light from the DUT, exiting the cryostat, is split into two arms. A fraction of the signal is incident upon an avalanche photodiode (APD) for broadband optical spectrum recording. The majority of the signal is passed to an optical spectrum analyzer (OSA, Yokogawa AQ6374) for characterizing the wavelengths and powers of generated sidebands when RF modulation is turned on. We measure the insertion losses and splitting ratios of the beam splitters immediately before and after the cryostat/DUT, thereby estimating losses induced by the cryostat optical ports and the couplings between the glued optical fibers and the DUT.

S4.2 Self-heterodyne measurement path

We characterize the optical modes, ω_k, independently using a self-heterodyne measurement, similar to those presented by Patel et al. [2] and Herrmann et al. [3]. The path for this measurement is depicted in Supplementary Figure 8.

For each optical mode, we feed back on the WM reading to lock the incident laser blue-detuned from resonance by a few GHz. With the Sercalo SL2x2 switch in cross configuration, light passes through a separate off-chip electro-optic intensity modulator. We modulate the EOM with the output from a

VNA (Rhode & Schwartz ZNB), thereby generating sidebands on the locked incident laser. We pass the pump, along with the sidebands, to the DUT. By sweeping the modulation frequency on the VNA, the red sideband from the EOM sweeps across the optical resonance. We detect the output pump light and both sidebands on a fast photodiode (F-PD, Optilab PD-40-M) and pass the resulting electrical signal, consisting of beat tones between the locked pump and swept sidebands, on the receiving port of the VNA. We normalize this spectrum to a VNA trace measured with the incident pump very far-detuned from resonance. Finally, we fit the result to an input-output model, from which we infer the parameters

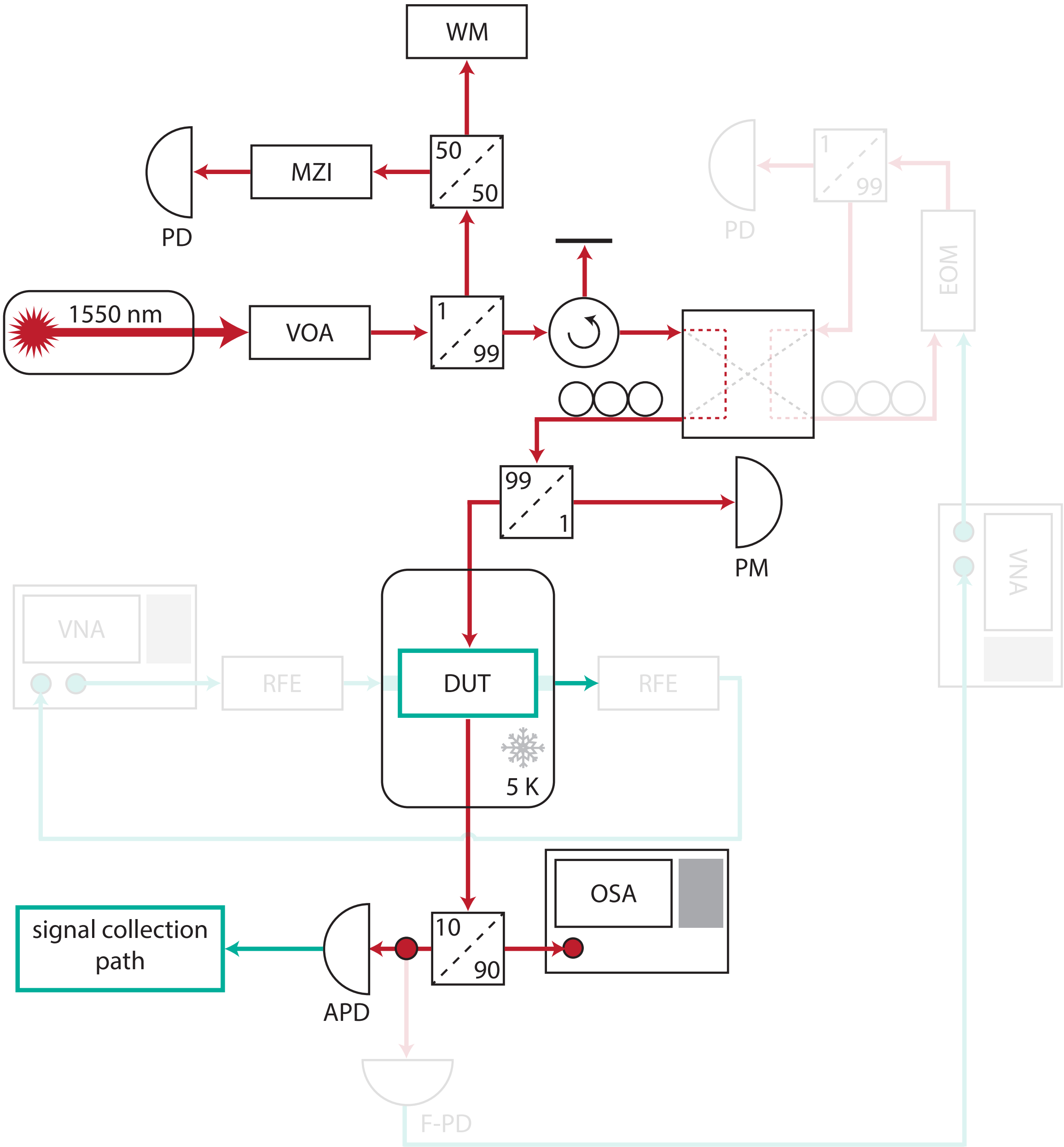

Supplementary Fig. 7 Primary optical path used in the transduction measurements. Calibrating the path losses and component losses (beam splitters, isolator, detectors), was critical in achieving self-consistent results. The acronyms are the same as in Supplementary Figure 6. The signal collection path is described further in Supplementary Figure 10.

of each optical mode. We present the results of this procedure applied to the optical pump mode ω_0, in main text Fig. 2b,c.

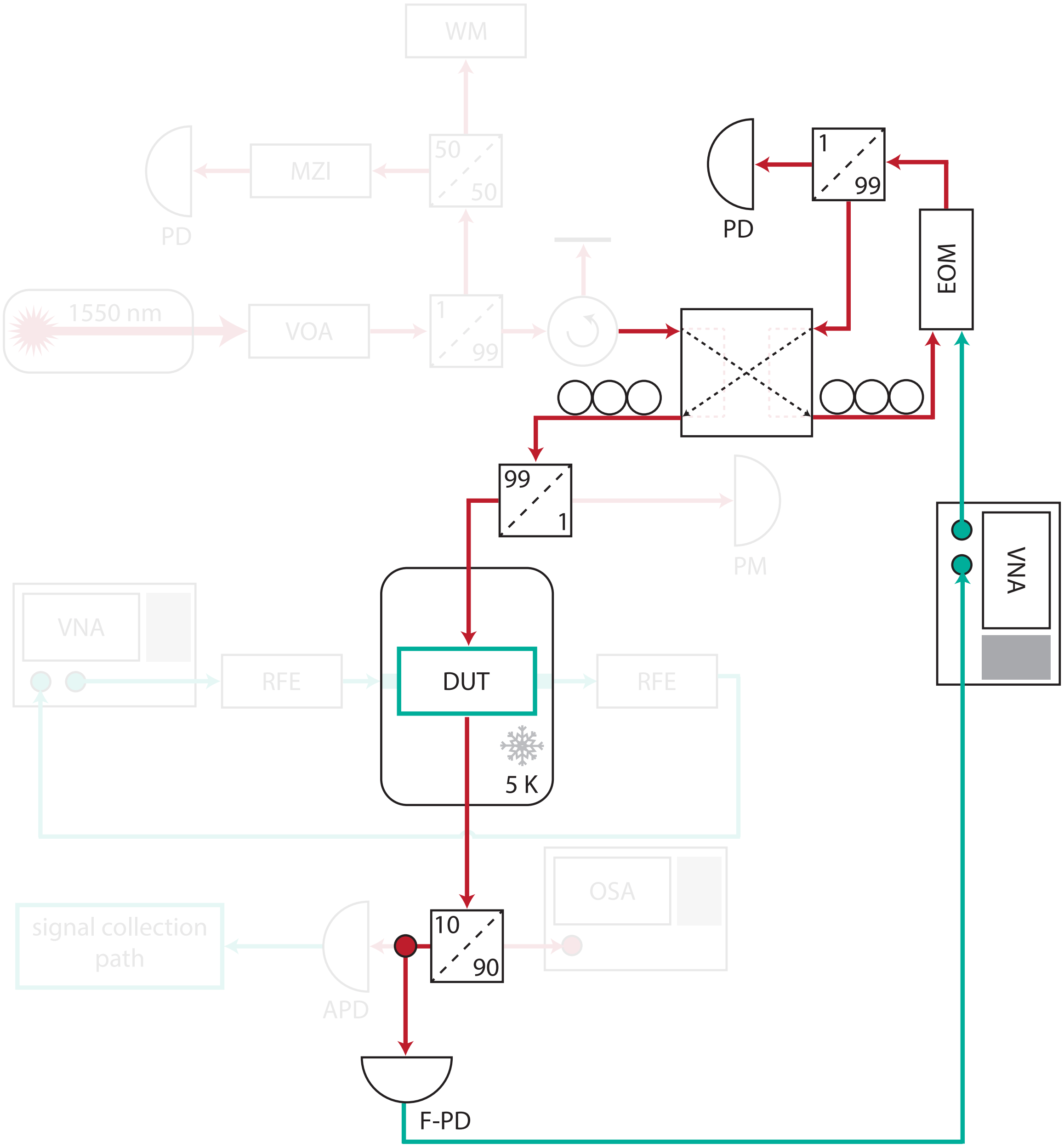

Supplementary Fig. 8 Self-heterodyne sub-path for optical mode characterization. Having the BNC switch, to switch between the primary optical path and this subsidiary path, enabled rapid phase-sensitive measurements of our optical modes. The acronyms are the same as in Supplementary Figure 6.

S4.2.1 Self-heterodyne measurement theory

In this section, we sketch the theoretical basis for the phase response measurement we performed to measure the optical cavity parameters discussed in the previous section. Consider the forward scattering matrix of a lossless, symmetric, and reciprocal Mach-Zehnder interferometer (MZI) [2–4],

$$\begin{pmatrix} \alpha_{\text{in}} \\ \cdot \end{pmatrix} = \left(\frac{1}{\sqrt{2}}\right)^2 \cdot \begin{pmatrix} 1 & i \\ i & 1 \end{pmatrix} \begin{pmatrix} 1 & 0 \\ 0 & e^{i\phi} \end{pmatrix} \begin{pmatrix} 1 & i \\ i & 1 \end{pmatrix} \cdot \begin{pmatrix} \alpha_{\text{L}} \\ 0 \end{pmatrix} \Longrightarrow \tag{S43}$$

$$\alpha_{\text{in}} = \frac{1}{2} \cdot \left(1 - e^{i\phi}\right) \cdot \alpha_{\text{L}}. \tag{S44}$$

Here ϕ is the relative phase delay in one arm of the MZI, α_{L} is the amplitude of the laser field, and α_{in} is the field we send to our optical cavity in the frame of the laser. In our case, we can vary the phase with a sinusoidal voltage at frequency Ω, so that $\phi = \phi(t) = \theta_{\text{DC}} + \beta\cos(\Omega t)$. This voltage signal comes from the source port of the VNA and is combined at the modulator with a DC voltage. Operating the modulator at mid-point so that $\theta_{\text{DC}} = \pi/2$ and assuming small modulation strength β we calculate,

$$\alpha_{\text{in}}(t) = \frac{1}{2} \cdot \left((1 - i) + \frac{\beta}{2}e^{-i\Omega t} + \frac{\beta}{2}e^{i\Omega t}\right) \cdot \alpha_{\text{L}}. \tag{S45}$$

We can write the frequency response of the optical cavity transmission in the frame of the source laser as,

$$\mathcal{T}(\omega; \Delta) = 1 - \frac{\kappa_e}{i(\Delta - \omega) + \kappa/2}, \tag{S46}$$

where $\Delta = \omega_c - \omega_{\text{L}}$, the detuning between the optical cavity mode frequency and the source laser frequency. Because the optical cavity is a linear time-invariant system we can write the transmitted field amplitude as (omitting 2Ω terms),

$$\alpha_{\text{trans}}(t; \Delta) = \frac{\alpha_{\text{L}}}{2} \cdot \left((1 - i)\mathcal{T}(0; \Delta) + \frac{\beta}{2}e^{-i\Omega t}\mathcal{T}(\Omega; \Delta) + \frac{\beta}{2}e^{i\Omega t}\mathcal{T}(-\Omega; \Delta)\right). \tag{S47}$$

The transmitted field is routed to a fast photodetector so that the beat frequency generates an RF signal that is AC-coupled to the receiving port of the VNA. Thus, the AC-coupled field is proportional to,

$$|\alpha_{\text{trans}}(t; \Delta)|^2 = \frac{|\alpha_{\text{L}}|^2\beta}{8} \cdot \left((1 + i)\mathcal{T}^*(0; \Delta)\mathcal{T}(\Omega) + (1 - i)\mathcal{T}(0; \Delta)\mathcal{T}^*(-\Omega; \Delta)\right) \cdot e^{-i\Omega t} \\ + \text{c.c.} \tag{S48}$$

On the VNA, we measure the Fourier transform of this expression, equivalent to the RF scattering parameter $S_{21}(\Omega)$ (i.e. the coefficient of the exponential). However, because this expression is proportional to the laser power ($|\alpha_{\text{L}}|^2$) and the RF power (β), this measurement includes optical path losses and the

frequency responses of the RF circuit elements. We normalize out these frequency-dependent losses by taking another measurement with the source laser detuned far away from the mode so that $\Delta \gg \kappa$. In this case, $\mathcal{T} \approx 1$ and we obtain:

$$\left|\alpha_{\text{trans}}^{\text{bg}}\right|^2 = \frac{|\alpha_\text{L}|^2 \beta}{4} \cdot e^{-i\Omega t} + \text{c.c.} \tag{S49}$$

Dividing the signal response by the far-detuned background, we obtain a normalized expression for the RF scattering parameter,

$$S_{21}^{\text{normalized}}(\Omega;\Delta) = \frac{1}{2}\Big((1+i)\mathcal{T}^*(0;\Delta)\mathcal{T}(\Omega) + (1-i)\mathcal{T}(0;\Delta)\mathcal{T}^*(-\Omega;\Delta)\Big). \tag{S50}$$

We use Eq. S50 and fit the phase response ($\angle S_{21}^{\text{normalized}}$) to deduce all the optical cavity parameters.

S4.3 Primary RF path

The primary RF modulation path in our experiment. The output signal from a specialized VNA (Rhode & Schwartz ZNA26) is up-converted through frequency mixing in a radio-frequency extender (RFE, OML Inc.) as highlighted in Supplementary Fig. 9. The RFE effectively translates the VNA range from $0-26$ GHz into $70-115$ GHz.

The RFE output is transitioned from WR10 waveguide into a 1 mm coaxial cable, which is fed into the cryostat via a hermetically sealed bulkhead connector. Inside the cryostat, the coaxial cable is translated back into a WR10 waveguide, enabling the RF signal to couple capacitively to the chip as explained in the main text. Transmission through the chip passes through an W-Band cryogenic isolator (Micro Harmonics), a W-Band cryogenic HEMT amplifier (Low Noise Factory LNF-LNC65_115WB), and another isolator (Micro Harmonics), before coupling back into 1 mm coaxial cable and routed out of the cryostat. This signal is down-converted to the native VNA range with a second RFE and recorded on the receiving port of the VNA.

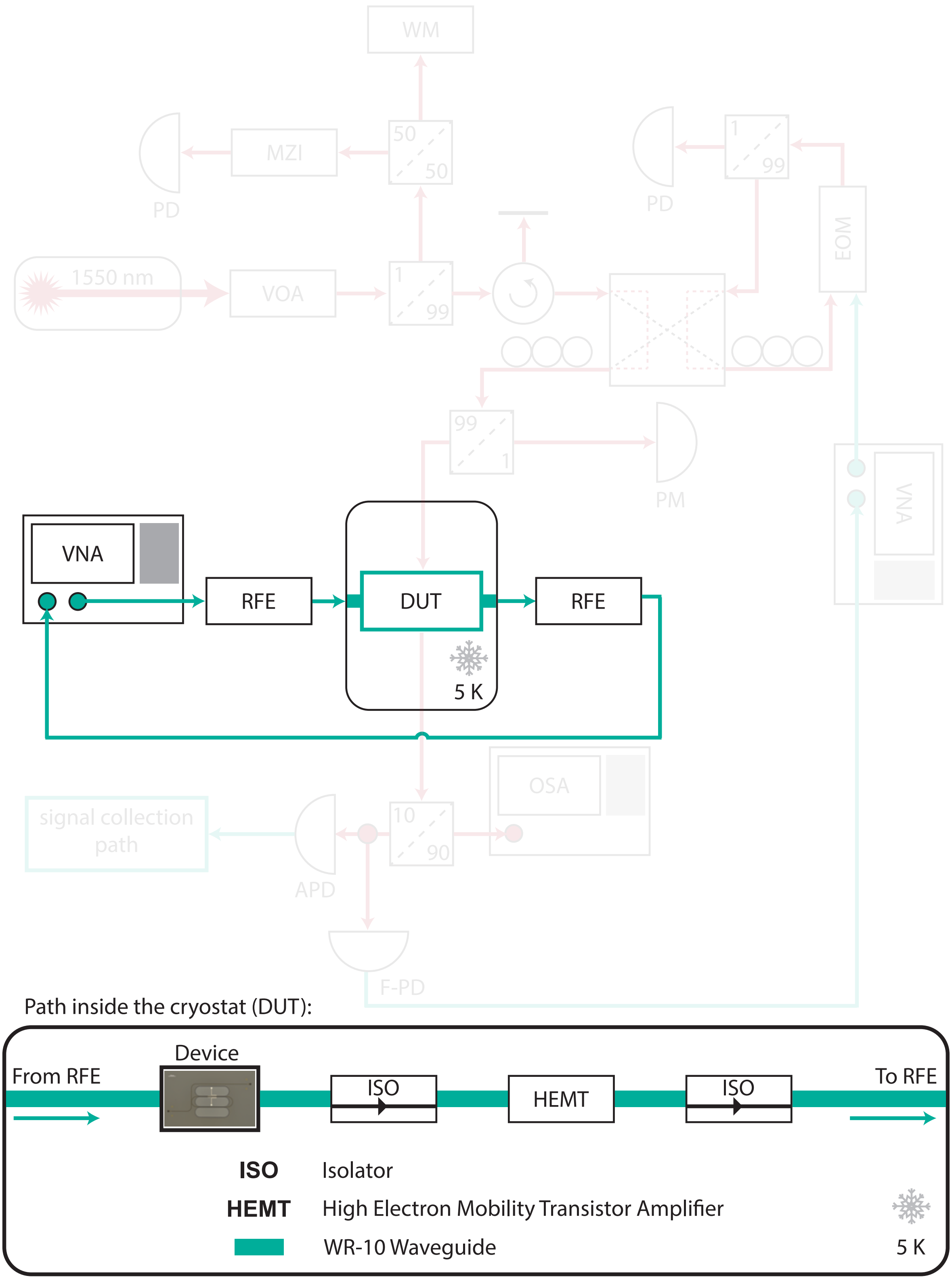

Supplementary Fig. 9 Primary RF path for on-chip mm-wave modulation. RFEs are used to up-/down-mix the VNA output into the range of 70 – 115 GHz. The signal passes through the device in transmission. The transmission passes through an isolator, a HEMT amplifier, and another isolator, before exiting the cryostat and being collected/recorded on the VNA.

S4.4 Optical-pump locking electronics path

The electronic sub-path used for optical pump locking is shown in Supplementary Figure 10. Before each transduction measurement, we must lock the incident laser in the pump mode ω_0, and calibrate its detuning from resonance. The mode drifts under locking due to thermal and photorefractive effects, so we lock slightly blue-detuned of the resonant frequency for increased stability.

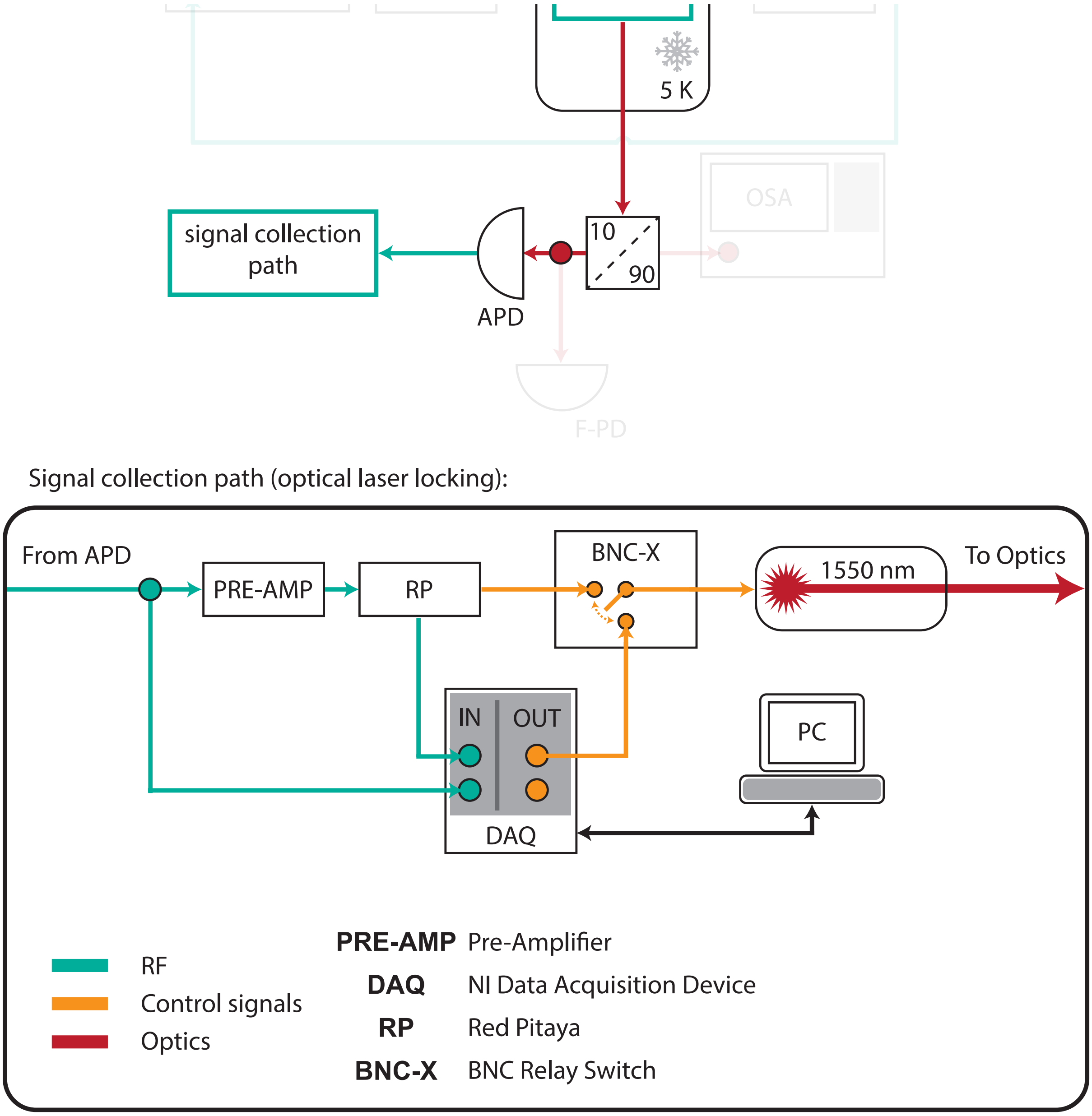

Supplementary Fig. 10 Electronic signal collection sub-path. This path is used for locking the incident pump laser to the optical mode, ω_0. The output of the APD is split into two paths, one recorded directly on a DAQ and PC, and the other passed through a pre-amp to the Red Pitaya voltage input. The output of the Red Pitaya and the DAQ are both used to modulate the laser, and a BNC mechanical relay switch is used to swap which output controls the laser.

S4.5 Images of packaging and cryostat

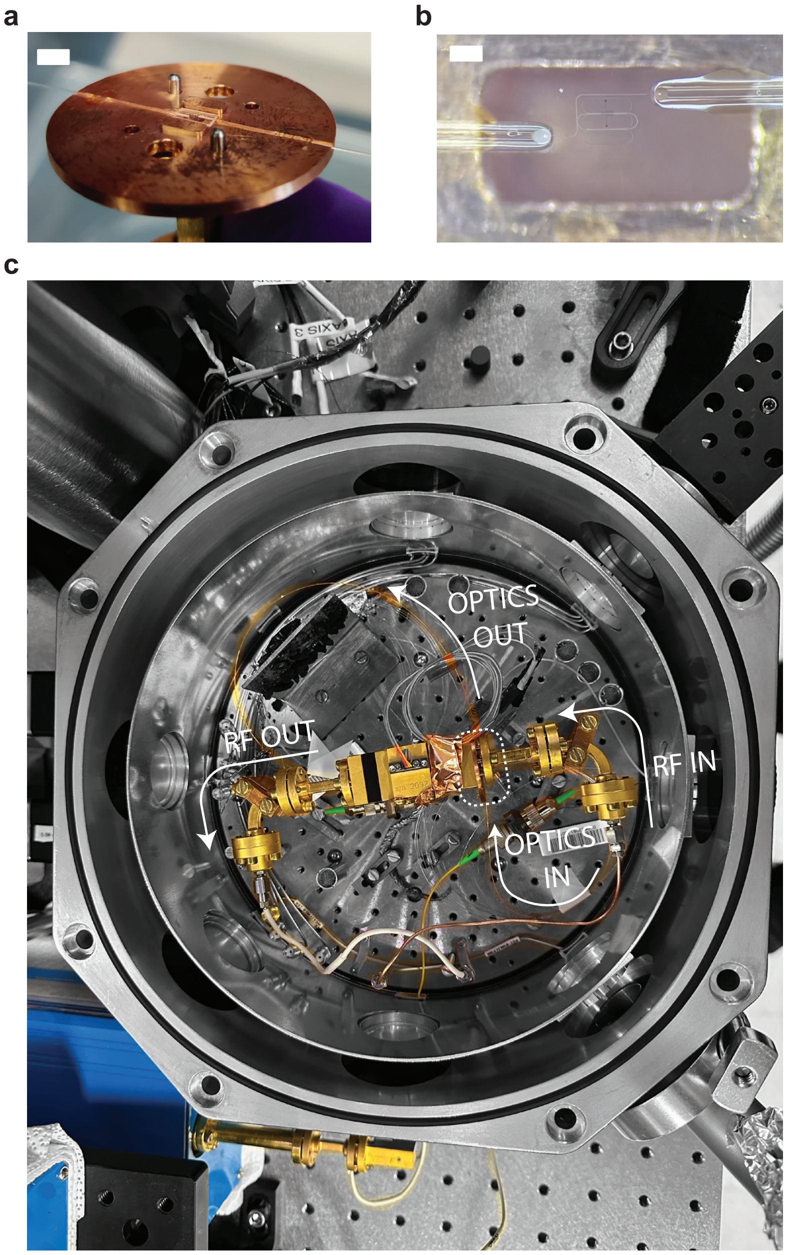

Supplementary Fig. 11 Images of the packaging and cryostat setup. **a** Smartphone image of the package device inside of the copper holder. Figure 1b in the main text is inspired by this image. The scale bar represents 5 mm. **b** Microscope image of the device immediately after the angle cleaved fibers were UV-cured onto the chip. The scale bar represents 300 µm. **c** Color-masked smartphone image of the cryostat preceding the cooldown. The regions with color emphasize the RF signal path and the optical signal path. The dashed box highlights where the device is fixed. Block diagrams of the RF and optical paths are attached in the Supplementary Information S4.

S5 Cryogenic photorefractive behavior and in-situ mitigation

During our first locking attempts at base temperature (∼4-5 K) we observed strong photorefractive effects, not uncommon in oxide-clad TFLN resonators [5]. Photo-excited charge carriers (from the optical pump) can distribute through the crystal, setting up a space-charge field that causes a refractive index change via the electro-optic effect. The practical impact of this in our measurements is that it limits how long our pump laser can remain locked to the optical pump mode. The maximum lock time is given by $t_{\mathrm{lock}} = (1\ \mathrm{V}) \cdot (dV_{\mathrm{piezo}}/dt)$, where 1 V is the maximum analog output from our Red Pitaya (0 V to 1 V).

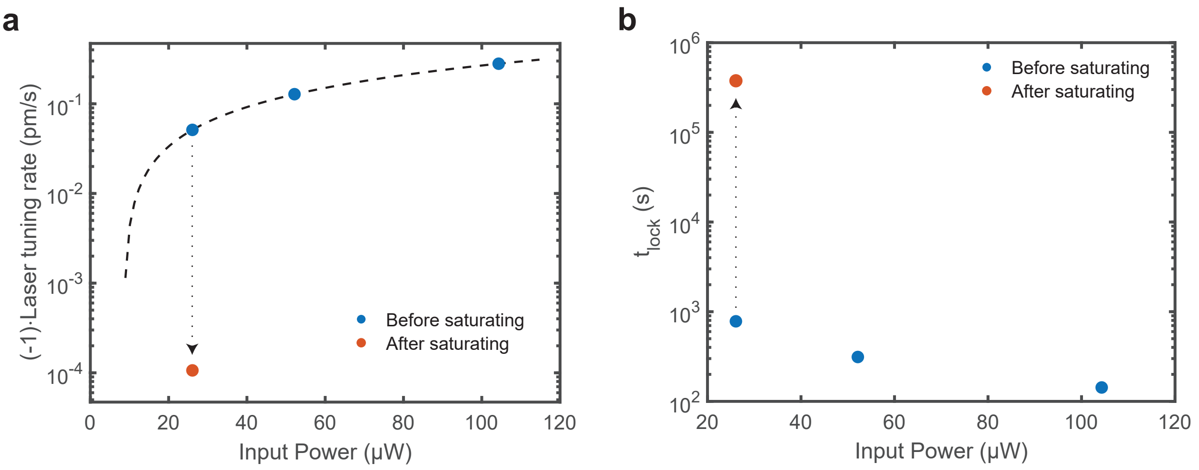

Supplementary Fig. 12 Drift due to photorefractive effect. **a** In this semi-log plot, we show the laser tuning rate (note, that this value is actually negative) as a function of optical power in the TFLN waveguide. The blue data show the drift rates before we reach steady-state (saturation). Near saturation, the drift rate reduces roughly 3 orders of magnitude, indicated by the dashed arrow and red data. The dashed line indicates a linear fit $y = (-2.9 \cdot 10^3\ (\mathrm{pm/s})/\mathrm{W}) \cdot P_{\mathrm{in}} + 0.03$ pm/s. **b** The corresponding data converted to the maximum lock time.

To quantify some of these effects, we measure the drift rate directly by looking at how fast the piezo-voltage set point changes from the Red Pitaya, while we are locked on a particular optical mode (see Sec. S4.4). The blue data in Supplementary Fig. 12a shows the laser tuning rate, $(dV_{\mathrm{piezo}}/dt) \cdot (-40\ \mathrm{pm/V})$, as a function of optical power. We can interpret this tuning rate as being equal to the cavity tuning rate, because we are locked at a fixed detuning from the cavity mode. The corresponding maximum lock time is shown in Supplementary Fig. 12b.

To saturate the effect, we lock to an optical mode with a power low enough that the lock time is on the order of 10 minutes. We repeat this until enough carriers are excited to stabilize the space-charge field. At this point, the lock time is much longer than we require to perform our electro-optic characterization. This procedure is indicated by the dashed arrow in Supplementary Fig. 12.

References

[1] Multani, K.K.S., Jiang, W., Nanni, E.A., Safavi-Naeini, A.H.: Quantum limits of superconducting-photonic links and their extension to millimeter waves. Physical Review Applied **22**(5), 054043 (2024) https://doi.org/10.1103/PhysRevApplied.22.054043

[2] Patel, R.N., Wang, Z., Jiang, W., Sarabalis, C.J., Hill, J.T., Safavi-Naeini, A.H.: Single-Mode Phononic Wire. Physical Review Letters **121**(4), 040501 (2018) https://doi.org/10.1103/PhysRevLett.121.040501

[3] Herrmann, J.F., Ansari, V., Wang, J., Witmer, J.D., Fan, S., Safavi-Naeini, A.H.: Mirror symmetric on-chip frequency circulation of light. Nature Photonics **16**(8), 603–608 (2022) https://doi.org/10.1038/s41566-022-01026-7

[4] Bucholtz, F., Singley, J.M.: Matrix representations for classical and quantum beam splitters. Optical Engineering **59**(12), 120801 (2020) https://doi.org/10.1117/1.OE.59.12.120801

[5] Xu, Y., Shen, M., Lu, J., Surya, J.B., Sayem, A.A., Tang, H.X.: Mitigating photorefractive effect in thin-film lithium niobate microring resonators. Optics Express **29**(4), 5497–5504 (2021) https://doi.org/10.1364/OE.418877

[6] COMSOL AB: COMSOL Multiphysics® v. 6.2. COMSOL AB **Stockholm, Sweden** (2024)

[7] Sonnet Software: Sonnet Suites Professional Release 19. Sonnet Software Inc. **Syracuse, NY** (2024)
